# Supplementary material for: Large language models can infer psychological dispositions of social media users
Source: PNAS Nexus. 2024 Jun 13;3(6):pgae231. doi: 10.1093/pnasnexus/pgae231 (PMC11211928; doi:10.1093/pnasnexus/pgae231)
Supplement: pgae231_Supplementary_Data [file pgae231_supplementary_data.pdf]

SUPPLEMENTARY INFORMATION LARGE LANGUAGE MODELS CAN INFER PSYCHOLOGICAL DISPOSITIONS OF SOCIAL MEDIA USERS

Heinrich Peters*
Columbia University
New York, USA
hp2500@columbia.edu

Sandra Matz
Columbia University
New York, USA
sm4409@columbia.edu

S1 Descriptive Statistics

		count	mean	std	min	25%	50%	75%	max
GPT-3.5	O	1000.0	3.074	0.364	1.933	2.833	3.100	3.333	4.133
	C	1000.0	2.177	0.347	1.133	1.967	2.100	2.333	3.800
	E	1000.0	3.401	0.511	1.833	3.058	3.467	3.800	4.500
	A	1000.0	2.581	0.637	1.000	2.133	2.567	3.033	4.500
	N	1000.0	3.056	0.499	1.900	2.667	3.067	3.408	4.400
GPT-4	O	1000.0	4.085	0.326	2.533	3.933	4.100	4.300	4.967
	C	1000.0	2.741	0.543	1.333	2.367	2.667	3.067	4.833
	E	1000.0	4.354	0.415	2.800	4.100	4.400	4.667	5.000
	A	1000.0	3.345	0.644	1.333	2.933	3.333	3.800	4.967
	N	1000.0	2.979	0.625	1.300	2.500	2.950	3.433	4.800
Self-report	O	1000.0	3.998	0.518	1.300	3.650	4.050	4.400	5.000
	C	1000.0	3.431	0.640	1.500	3.000	3.450	3.900	5.000
	E	1000.0	3.473	0.766	1.100	2.950	3.550	4.050	5.000
	A	1000.0	3.613	0.589	1.300	3.250	3.650	4.050	5.000
	N	1000.0	2.742	0.779	1.000	2.200	2.700	3.250	4.700

Table S1. Descriptive statistics for Big Five personality scores by GPT-3.5, GPT-4, and self-report (IPIP). O: Openness; C: Conscientiousness; E: Extraversion; A: Agreeableness; N: Neuroticism.

S2 Correlation Analyses

		cor	ci_l	ci_u	p
GPT-3.5	O	0.282	0.224	0.338	0.0
	C	0.223	0.163	0.281	0.0
	E	0.291	0.233	0.346	0.0
	A	0.298	0.240	0.353	0.0
	N	0.263	0.205	0.320	0.0
GPT-4	O	0.327	0.270	0.381	0.0
	C	0.264	0.206	0.321	0.0
	E	0.324	0.268	0.379	0.0
	A	0.325	0.268	0.379	0.0
	N	0.294	0.236	0.349	0.0

Table S2. Correlations between inferred and self-reported personality scores for GPT-3.5 and GPT-4 with two-tailed 95% confidence intervals and p-values. O: Openness; C: Conscientiousness; E: Extraversion; A: Agreeableness; N: Neuroticism.

S3 Correlations as a Function of Input Volume

chunk	messages	O	C	E	A	N
1	20.0	0.220	0.176	0.225	0.221	0.201
2	40.0	0.223	0.207	0.244	0.247	0.208
3	60.0	0.248	0.211	0.261	0.259	0.230
4	80.0	0.260	0.206	0.266	0.276	0.240
5	100.0	0.271	0.215	0.272	0.293	0.263
6	120.0	0.270	0.217	0.272	0.294	0.265
7	140.0	0.276	0.219	0.272	0.290	0.265
8	160.0	0.284	0.216	0.283	0.289	0.260
9	180.0	0.279	0.217	0.286	0.297	0.263
10	200.0	0.281	0.222	0.290	0.298	0.263

Table S3.1. Correlations as a function of input volume for inferences by GPT-3.5. O: Openness; C: Conscientiousness; E: Extraversion; A: Agreeableness; N: Neuroticism.

chunk	messages	O	C	E	A	N
1	20.0	0.225	0.219	0.219	0.257	0.233
2	40.0	0.274	0.265	0.256	0.281	0.263
3	60.0	0.276	0.261	0.281	0.290	0.283
4	80.0	0.290	0.257	0.299	0.309	0.272
5	100.0	0.293	0.253	0.308	0.318	0.282
6	120.0	0.292	0.254	0.312	0.321	0.290
7	140.0	0.290	0.253	0.310	0.323	0.292
8	160.0	0.306	0.255	0.314	0.321	0.294
9	180.0	0.320	0.259	0.320	0.325	0.295
10	200.0	0.327	0.264	0.324	0.325	0.294

Table S3.2. Correlations as a function of input volume for inferences by GPT-4. O: Openness; C: Conscientiousness; E: Extraversion; A: Agreeableness; N: Neuroticism.

S4 Subgroup Analysis - Gender

		GPT-3.5			GPT-4		
		d	t	p	d	t	p
**Self-report scores**	O	-0.067	-1.022	-0.307	-0.067	-1.022	-0.307
	C	-0.038	-0.578	-0.563	-0.038	-0.578	-0.563
	E	-0.022	-0.336	-0.737	-0.022	-0.336	-0.737
	A	0.151	2.309	-0.021	0.151	2.309	-0.021
	N	0.428	6.530	-0.000	0.428	6.530	-0.000
**Inferred scores**	O	0.224	3.419	-0.001	0.179	2.724	-0.007
	C	0.346	5.280	-0.000	0.375	5.729	-0.000
	E	0.342	5.214	-0.000	0.475	7.250	-0.000
	A	0.887	13.530	-0.000	0.894	13.634	-0.000
	N	-0.056	-0.861	-0.389	0.060	0.917	-0.360
**Absolute residuals**	O	-0.251	-3.827	-0.000	-0.060	-0.921	-0.357
	C	-0.232	-3.534	-0.000	-0.294	-4.481	-0.000
	E	-0.089	-1.363	-0.173	0.208	3.181	-0.002
	A	-0.605	-9.224	-0.000	-0.342	-5.218	-0.000
	N	-0.298	-4.553	-0.000	-0.157	-2.394	-0.017
**Directed residuals**	O	0.214	3.270	-0.001	0.181	2.762	-0.006
	C	0.219	3.335	-0.001	0.315	4.811	-0.000
	E	0.242	3.690	-0.000	0.284	4.331	-0.000
	A	0.618	9.428	-0.000	0.641	9.786	-0.000
	N	-0.450	-6.864	-0.000	-0.347	-5.300	-0.000

Table S4. Comparisons of self-report scores, inferred scores, absolute residuals, and directed residuals across gender groups. A positive test statistic indicates a higher group mean for female users. O: Openness; C: Conscientiousness; E: Extraversion; A: Agreeableness; N: Neuroticism.

S5 Subgroup Analysis - Age

		GPT-3.5			GPT-4		
		d	t	p	d	t	p
**Self-report scores**	O	0.198	2.961	-0.003	0.198	2.961	-0.003
	C	0.487	7.276	-0.000	0.487	7.276	-0.000
	E	0.050	0.751	-0.453	0.050	0.751	-0.453
	A	0.088	1.312	-0.190	0.088	1.312	-0.190
	N	-0.220	-3.283	-0.001	-0.220	-3.283	-0.001
**Inferred scores**	O	-0.192	-2.860	-0.004	0.048	0.720	-0.472
	C	0.618	9.228	-0.000	0.697	10.414	-0.000
	E	-0.237	-3.546	-0.000	-0.024	-0.363	-0.717
	A	0.326	4.867	-0.000	0.294	4.388	-0.000
	N	-0.230	-3.429	-0.001	-0.292	-4.368	-0.000
**Absolute residuals**	O	0.320	4.777	-0.000	-0.017	-0.253	-0.801
	C	0.177	2.638	-0.008	0.052	0.769	-0.442
	E	0.080	1.201	-0.230	-0.077	-1.150	-0.251
	A	-0.177	-2.636	-0.009	0.076	1.140	-0.255
	N	-0.001	-0.010	-0.992	0.009	0.132	-0.895
**Directed residuals**	O	-0.318	-4.750	-0.000	-0.171	-2.548	-0.011
	C	-0.152	-2.266	-0.024	0.071	1.064	-0.288
	E	-0.203	-3.031	-0.003	-0.066	-0.979	-0.328
	A	0.212	3.161	-0.002	0.192	2.860	-0.004
	N	0.072	1.080	-0.280	-0.011	-0.169	-0.866

Table S5. Comparisons of self-report scores, inferred scores, absolute residuals, and directed residuals across age groups. A positive test statistic indicates a higher group mean for users of above-median age. O: Openness; C: Conscientiousness; E: Extraversion; A: Agreeableness; N: Neuroticism.

S6 Agreement With Third-Person Observer Ratings

S6.1 Observer Ratings and Self-Reports

	cor	ci_l	ci_u	p
O	0.352	-0.005	0.447	0.055
C	0.198	0.179	0.583	0.001
E	0.334	-0.089	0.377	0.214
A	0.378	0.174	0.580	0.001
N	0.261	-0.045	0.414	0.111

Table S6.1. Correlations between third-person observer ratings and self-reported personality scores with two-tailed 95% confidence intervals and p-values. O: Openness; C: Conscientiousness; E: Extraversion; A: Agreeableness; N: Neuroticism.

S6.2 Observer Ratings and LLM Inferences

		cor	ci_l	ci_u	p
GPT-3.5	O	0.284	0.049	0.489	0.019
	C	0.457	0.246	0.627	0.000
	E	0.057	-0.184	0.292	0.643
	A	0.396	0.175	0.580	0.001
	N	0.153	-0.089	0.377	0.214
GPT-4	O	0.234	-0.005	0.447	0.055
	C	0.400	0.179	0.583	0.001
	E	0.152	-0.089	0.377	0.214
	A	0.396	0.174	0.580	0.001
	N	0.195	-0.045	0.414	0.111

Table S6.2. Correlations between third-person observer ratings and LLM-inferred personality scores with two-tailed 95% confidence intervals and p-values. O: Openness; C: Conscientiousness; E: Extraversion; A: Agreeableness; N: Neuroticism.
